# Process Optimization of Novel Boronophenylalanine Liposomes Through Box–Behnken Response Surface Design and Preliminary Evaluation in A549 Lung Carcinoma Cells for Boron Neutron Capture Therapy

**DOI:** 10.3390/molecules31091409

**Published:** 2026-04-24

**Authors:** Haojie Shi, Qianlong Xu, Fenglin Li, Caiyun Fan, Yi Han

**Affiliations:** China Institute of Atomic Energy, Beijing 102400, China; m13898215096@163.com (Q.X.); 13522048467@163.com (C.F.); yihan9821@163.com (Y.H.)

**Keywords:** CB-BPA-Lips, liposome, BNCT, BPA, o-carborane, BBD-RSM, A549 cell lines, lung cancer

## Abstract

Boron neutron capture therapy (BNCT) is a binary targeted radiotherapy that uses boron agents to treat refractory malignancies. This study developed a novel boronophenylalanine (BPA)-loaded liposome doped with o-carborane (CB) for BNCT. We applied response surface methodology (RSM) to identify factors affecting BPA loading and optimized encapsulation efficiency (EE) to minimize BPA loss. In in vitro experiments, these liposomes demonstrated promising characteristics for BNCT. The nanoparticle properties of CB-BPA-Lips remain stable for at least 48 h, and CB-BPA-Lips can effectively reduce the release of the agents loaded within them. Both cell viability assays and apoptosis assays have shown that CB-BPA-Lips have good biocompatibility and a lower inhibitory effect on cell viability than BPA. Cellular boron uptake peaked at 47.3642 ng B/10^6^ cells in A549 lung cancer cells and peaked at 38.8875 ng B/10^6^ cells in Bronchial Epithelium transformed with Ad12-SV40 2B (BEAS-2B) human normal bronchial epithelial cells at 24 h post-treatment, with both exceeding uptake in the BPA control group. Overall, this work presents an optimized liposomal formulation that enhances boron delivery to cancer cells and provides a potential candidate boron agent for BNCT pending in-depth in vivo studies.

## 1. Introduction

Based on tumor-specific delivery of boron-10 and thermal neutron irradiation, boron neutron capture therapy (BNCT) is a tumor cell-specific radiotherapy using ^10^B(n,α)^7^Li nuclear reaction [1,2,3,4,5,6]. One of the key factors for the success of BNCT lies in the selection of boron agents. Currently, boron agents are mainly classified into three generations. Among them, the second-generation boron carriers, sodium borocaptate (Na_2_^10^B_12_H_11_SH, BSH) and boronophenylalanine (BPA), have been extensively studied, and promising results have been achieved. However, the further development of second-generation boron agents has been limited by high administration doses (which can increase treatment costs), limited tumor targeting, short in vivo circulation, or brief intratumoral retention.

Liposomes (Lips) are nanoscale delivery vehicles with a phospholipid bilayer structure with hydrophilic and hydrophobic compounds co-encapsulated in their aqueous core and membrane [7]. Lips can selectively accumulate in tumor tissues by utilizing the enhanced permeability and retention (EPR) effect, which takes advantage of the leaky vasculature and inadequate lymphatic drainage typical of solid tumors [8]. Clinically approved antitumor liposomes—far more numerous than other nano-drug delivery systems—offer a promising platform for delivering boron agents for BNCT because of their excellent biocompatibility and biodegradability. It has been successfully used for refractory head and neck tumors, high-grade gliomas, and melanomas. Its potential, however, extends beyond glioma, leaving substantial opportunity to explore BNCT for other common malignancies. Lung adenocarcinoma, the most frequent subtype of non-small cell lung cancer, comprises approximately 40% to 50% of lung cancer cases and ranks among the world’s most prevalent malignancies [9,10]; it presents insidiously, progresses rapidly, and poses a serious threat to public health. Current clinical management of lung adenocarcinoma uses a spectrum of modalities, including surgery, chemotherapy, radiotherapy, targeted therapy, and immunotherapy. These conventional approaches, however, commonly induce irreversible injury to normal lung tissue and impair pulmonary function [11,12,13]. BNCT, an emerging form of precision radiotherapy, can minimize such collateral damage while treating tumors. By preserving pulmonary function, BNCT may improve post-treatment quality of life and offer a promising direction for more precise management of lung adenocarcinoma. Numerous studies encapsulate BSH or BSH derivatives within liposomes to improve tumor delivery; compared with free BSH, these formulations require lower doses and increase boron uptake by tumor cells [14,15,16]. However, BSH has not received any permission for marketing approval, and the in vivo sustained release of agents encapsulated in the hydrophilic phase of liposomes is nearly unavoidable. After the administration of BSH-Lips, the free BSH released in the non-tumor site lacks the ability to effectively target tumors. The tumor-targeting molecule BPA, which is the approved boron agent for surgically unresectable locally advanced or locally recurrent head and neck cancers (approved in Japan) [7,17,18], poses challenges such as poor pharmacokinetics characterized by a short half-life and high dosing requirements (~500 mg/kg). Research on BPA-Lips deserves significant attention. This may lay the foundation for achieving long-term high boron concentration in tumor sites after a single intravenous administration to patients in the future.

**Figure 1:**
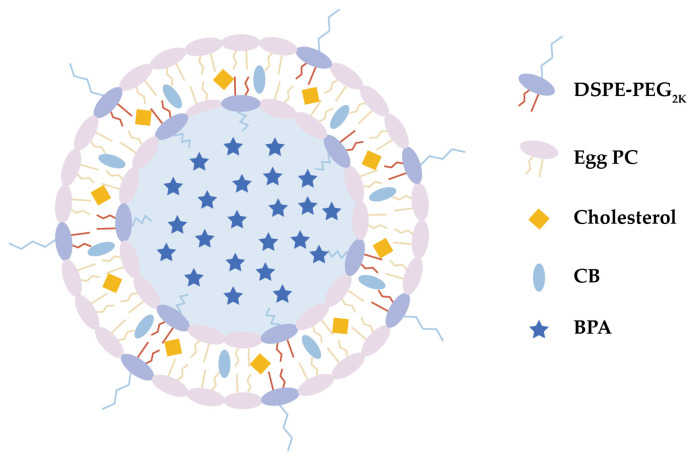
Structure of CB-BPA-Lips. DSPE, 1,2-distearoyl-sn-glycero-3-phosphoethanolamine; PEG, polyethylene glycol; PC, phosphatidylcholine; CB, o-carborane; BPA, boronophenylalanine; and Lip, liposome.

Hence, this study employed liposomes to encapsulate BPA, and the preliminary research results of the novel BPA liposomes in lung adenocarcinoma cells were reported. To enhance the boron payload and optimize vesicle space utilization, boron-rich small molecules, o-carborane (CB), were incorporated into the phospholipid bilayer. This approach resulted in the development of a BPA-loaded liposomal delivery system, referred to as CB-BPA-Lips (Figure 1). Additionally, the CB-BPA-Lips formulation was optimized for boronophenylalanine (BPA) encapsulation efficiency (EE). Initial single-factor experiments evaluated key parameters: the mass ratio of phospholipid to cholesterol, the mass ratio of phospholipid to CB, the mass ratio of phospholipid to BPA, and hydration time. These parameters were then refined by a Box–Behnken design (BBD) and response surface methodology (RSM) to maximize BPA loading [19,20,21]. In vitro release assay characterized the release profile of small molecules in the CB-BPA-Lips. In vitro serum stability assay assessed maintenance of physicochemical properties over time. A549 human lung adenocarcinoma cells were selected because they are a well-established and widely used model of lung adenocarcinoma. This cell line reliably reproduces key features of the disease, making it a valuable tool for testing potential treatments and probing disease mechanisms. To eliminate the potential influence of BPA released from CB-BPA-Lips on the experimental outcomes, the LAT1-negative BEAS-2B human normal bronchial epithelial cell line was employed to replicate the cellular experiments. Cell viability assay of cells compared the half-maximal inhibitory concentration (IC_50_) of free BPA versus CB-BPA-Lips. Intracellular boron accumulation from free BPA, BPA-Lips, and CB-BPA-Lips was quantified by cellular uptake analysis. The apoptosis caused by CB-BPA-Lips was studied by flow cytometry, aiming to explore whether they exhibit comparable biocompatibility by comparing the apoptosis promoted by CB-BPA-Lips with that promoted by BPA. The findings of this study may contribute to the management of lung cancer via BNCT, providing a strong basis for further investigations.

## 2. Results

### 2.1. Optimization of the BPA EE by RSM

#### 2.1.1. Single-Factor Experimental Analysis

A single-factor exploration is essential prior to implementing BBD-based optimization to identify the influencing factors and delineate the scope. Consequently, the following exploration was conducted.

**Figure 2:**
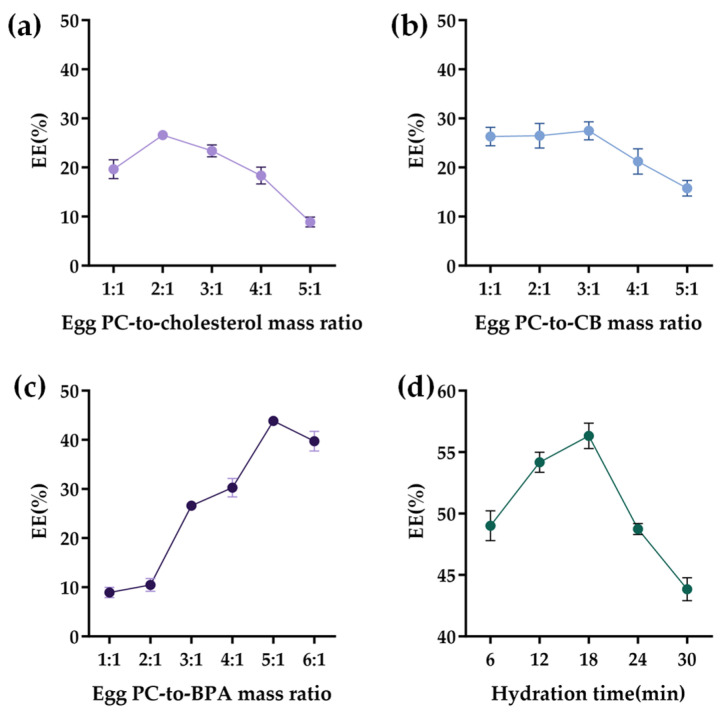
The effect of single factors on the BPA EE. (**a**) Egg PC-to-cholesterol mass ratio; (**b**) Egg PC-to-CB mass ratio; (**c**) Egg PC-to-BPA mass ratio; and (**d**) hydration time. EE, encapsulation efficiency. All data are an average of three independent experiments.

The BPA EE increased dose-dependently with an increase in the Egg PC-to-cholesterol mass ratio up to 2:1, after which it declined (Figure 2a). This biphasic behavior was attributed to the role of cholesterol in regulating the packing density and permeability of the lipid bilayer.

Experiments showed that the BPA EE did not increase significantly as the Egg PC-to-CB mass ratio rose from 1:1 to 3:1. When the ratio exceeded 3:1, the BPA EE fell markedly (Figure 2b). This decline likely reflects a limit to liposomal loading capacity, with additional CB in the formulation interfering with the BPA EE.

The relationship of the Egg PC-to-BPA mass ratio and the BPA EE revealed that the EE increased sharply with ratios ranging from 1:1 to 5:1 and abruptly decreased at higher ratios (Figure 2c). This finding suggests that an increase in Egg PC significantly improves the efficiency of liposome encapsulation of BPA. However, when the phospholipid concentration exceeds optimal levels, the viscosity of the system rises, leading to a self-assembly process that is more likely to produce non-bilayer structures or multilayer vesicle stacking. Such abnormal structures possess a limited capacity for encapsulating water-soluble drugs, ultimately resulting in a decreased encapsulation rate.

The BPA EE also exhibited a non-monotonic dependence on hydration time (Figure 2d), sharply increasing and peaking at 18 min, followed by a subsequent decline with longer hydration times, which was attributable to structural compromise from prolonged exposure.

Initial single-factor experiments selected the influencing factors that significantly affect the enhancement of the BPA EE, and the optimization range was defined. Then, a BBD response surface methodology was further employed to optimize the BPA EE. Based on the above results, the following ranges were selected for further RSM optimization: Egg PC-to-cholesterol mass ratio, 1:1–3:1; Egg PC-to-BPA mass ratio, 4:1–6:1; and hydration time, 12–24 min.

#### 2.1.2. BBD-Based Optimization

A total of 17 experiments were designed through BBD and performed in triplicate (Table 1). Multiple regression analysis was performed using the quadratic polynomial model described by Equation (1), linking the independent variables to EE response:

(1) $$Y=56.32+0.5366X_{1}+1.73X_{2}-0.2654X_{3}-0.4980X_{1}X_{2}+0.5468X_{1}X_{3}+0.0390X_{2}X_{3}-8.95X_{1}^{2}-6.52X_{2}^{2}-5.14X_{3}^{2}$$

Analysis of variance for the regression model (Table 2) revealed that the model exhibited a highly significant F-value of 830.28 (*p* = 0.0001). The determination coefficient R^2^ = 0.9991 was considerably high and indicated that 99.91% of the variability was explained, and only 0.09% was unexplained. The adjusted determination coefficient R_adj_^2^ = 0.9979 is closely associated with R^2^, demonstrating excellent model fit and agreement with the experimental results. Additionally, the lack-of-fit test revealed nonsignificant results (*p* = 0.4455), confirming that the model satisfactorily describes the observed data. Among the model terms, the linear coefficient of X_1_, quadratic coefficients (X_1_^2^, X_2_^2^, and X_3_^2^), and two interaction terms (X_1_X_2_ and X_1_X_3_) significantly affected EE (*p* < 0.05). In contrast, the linear coefficients of X_2_ and X_3_, along with the interaction term X_2_X_3_, exhibited nonsignificant effects (*p* > 0.05). The low coefficient of variation (0.6679%) further reflected the high precision and reliability of the experimental data used for model fitting.

#### 2.1.3. Response Surface Analysis

**Figure 3:**
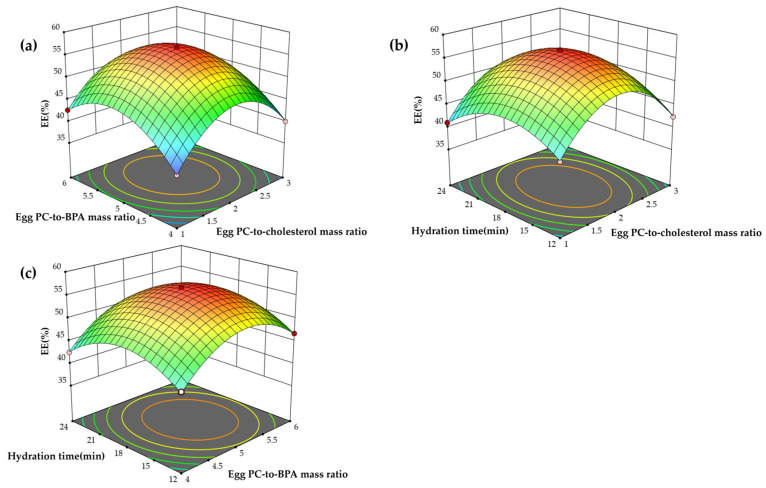
The correlation results between the variables highlighted by three-dimensional response surface plots. (**a**) The correlation results between the Egg PC-to-cholesterol mass ratio and Egg PC-to-BPA mass ratio; (**b**) the correlation results between the Egg PC-to-cholesterol mass ratio and hydration time; and (**c**) the correlation results between the Egg PC-to-BPA mass ratio and hydration time.

The interactive effects between the independent variables on the BPA EE are visualized in the 3D response surface plots (Figure 3a–c). The convex nature of all surfaces confirms the appropriateness of the defined variable ranges. Specifically, Figure 3a reveals that while EE initially benefited from concurrent increases in the mass ratios of Egg PC to cholesterol (X_1_) and Egg PC to BPA (X_2_), a reversal was observed at higher X_2_ levels under elevated X_1_ conditions, suggesting a saturation threshold. Figure 3b portrays the interaction between X_1_ and hydration time (X_3_), characterized by an initial rise and subsequent fall in EE, indicative of an optimal processing window. Figure 3c demonstrates that maximum EE was attained at about 18 min of hydration and about a 5:1 Egg PC-to-BPA ratio, with performance declining thereafter, possibly as a result of membrane rearrangement or drug leakage under extended hydration. Using a 3D response surface method, we found that an Egg PC-to-cholesterol mass ratio of 2.026:1, an Egg PC-to-BPA mass ratio of 5.131:1, and a hydration time of 17.853 min maximized BPA encapsulation at 56.445%. The measured value of 57.644 ± 0.21% (*n* = 3) closely matched the model prediction, confirming the accuracy of the model and its applicability to future scale-up.

### 2.2. Characterization

**Figure 4:**
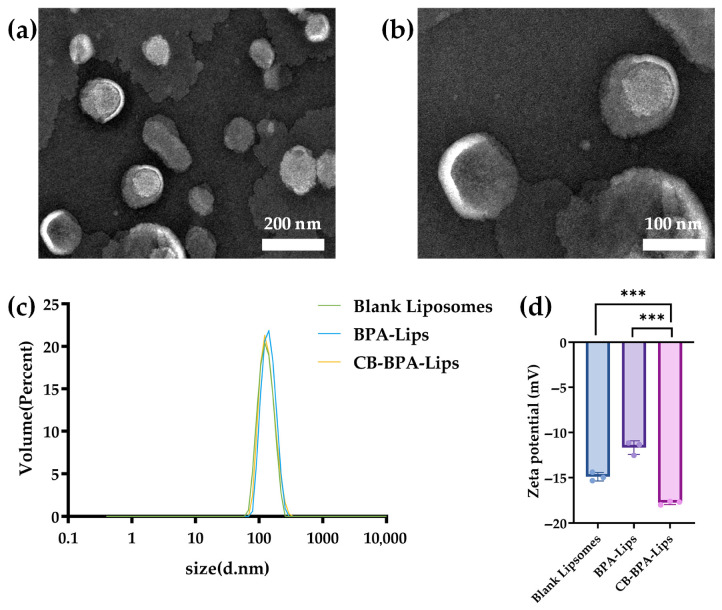
Characterization. (**a**) Transmission electron microscopy of CB-BPA-Lips (scale bar: 200 nm); (**b**) transmission electron microscopy of CB-BPA-Lips (scale bar: 100 nm); (**c**) hydrodynamic diameters of CB-BPA-Lips; and (**d**) zeta potential of blank liposomes, BPA-Lips, and CB-BPA-Lips. All data are an average of three independent experiments. *** *p* ≤ 0.001.

The physicochemical characteristics of CB-BPA-Lips are summarized in Figure 4. The Lips exhibited a hydrodynamic diameter of 146 ± 13 nm, with a narrow distribution ranging from 70 to 300 nm and a polydispersity index (PDI) of 0.170 ± 0.036, which confirmed a highly uniform dispersion suitable for drug delivery. Additionally, the zeta potential of blank liposomes, BPA-Lips, and CB-BPA-Lips was measured to confirm the loading of BPA and CB, which were −14.90 ± 0.46 mV, −11.68 ± 0.74 mV, and −17.77 ± 0.21 mV, respectively. The small change in zeta potential between BPA-Lips and blank liposomes indicates that BPA is encapsulated in the liposomal aqueous core, while the additional shift observed for CB-BPA-Lips relative to BPA-Lips reflects CB insertion into the lipid bilayer. These results collectively confirm successful loading of both BPA and CB in the liposomes.

### 2.3. In Vitro Serum Stability Assay

Lips, as injectable nano-drug delivery systems, are rapidly exposed to a high-abundance protein environment after entering the bloodstream. The 50% fetal bovine serum (FBS, Beyotime Biotech Inc., Shanghai, China) was used in an in vitro system to simulate the blood environment for evaluating the stability of CB-BPA-Lips. This well-established model has been validated by multiple studies for assessing in vitro liposome stability. Using a high FBS concentration can prevent artefactual stability that might arise when low-concentration FBS causes only negligible property changes to Lips.

**Figure 5:**
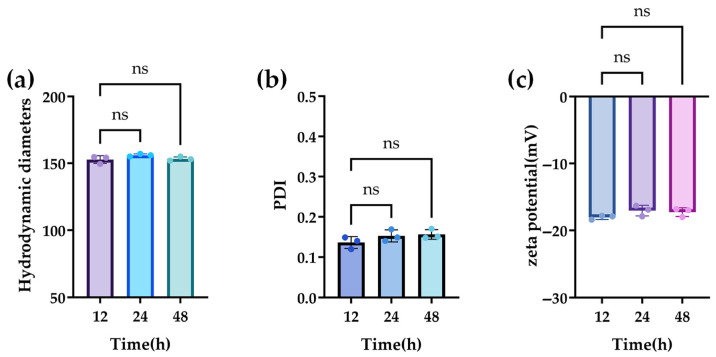
In vitro serum stability of the prepared CB-BPA-Lips. The CB-BPA-Lips were incubated with 50% FBS for 12, 24, and 48 h. (**a**) Hydrodynamic diameters; (**b**) PDI; and (**c**) zeta potential. PDI, polydispersity index. “ns” stands for not significant. All data are an average of three independent experiments.

The stability of the properties of CB-BPA-Lips was investigated in serum over 48 h. Ultimately, the nearly unchanged curves in the hydrodynamic diameter, PDI, and zeta potential demonstrate the prolonged stability of CB-BPA-Lips in 50% bovine serum at 37 °C (Figure 5).

### 2.4. In Vitro Release Assay

**Figure 6:**
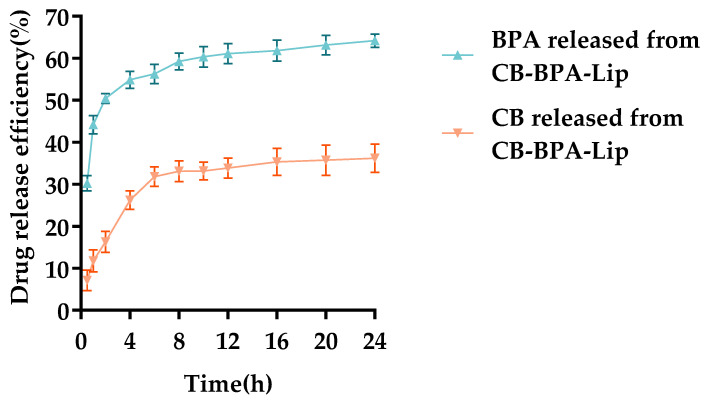
In vitro release curve. All data are an average of three independent experiments.

Studying drug release from liposomes is essential to assess whether they have the potential to decelerate release, prolong drug circulation, and deliver payloads to tumors. To evaluate the sustained-release behavior of CB-BPA-Lips in vitro, dialysis release profiles of CB-BPA-Lips were measured in PBS (pH = 7.4) containing 10% Tween 80. As shown in Figure 6, only about 55% of the BPA from CB-BPA-Lips was released within 8 h (the released BPA still retains the ability to actively target tumors). Release of CB from the liposomes was likewise markedly slower.

Establishing mathematical models is an effective tool for studying the drug release kinetics of drug-loaded nanoparticles. The most commonly used mathematical models are employed to study drug release kinetics, including the zero-order model, the first-order model, the Weibull model, the Higuchi model, and the Korsmeyer–Peppas model. These models fit the data through linear regression, with R^2^ as the coefficient of determination. The fitting parameters are shown in Table 3. For the BPA loaded in CB-BPA-Lips, the zero-order model, the first-order model, the Higuchi model, and the Korsmeyer–Peppas model cannot well describe the release kinetics of BPA (according to R^2^). However, the release process can be well described by the Weibull model (R^2^ = 0.9934). According to β < 1 (β is the curve shape parameter), it can be known that the release process of BPA from CB-BPA-Lips first experiences a burst release effect, followed by a release plateau. This is a common phenomenon of the release of water-soluble drugs loaded in liposomes due to the osmotic pressure difference between the internal and external aqueous phases, and it also indicates that there is no rupture of the liposome membrane or liposome aggregation during the entire release process. For the CB loaded in CB-BPA-Lips, both the first-order model and the Weibull model can better explain its release kinetics. Moreover, the fact that β = 0.9408 in the Weibull model is close to 1 also corroborates that the CB release process fits well with the results of the first-order kinetic model. Both models indicate that CB is released through diffusion across the intact phospholipid bilayer. The release of CB also experiences the burst release effect. However, since CB is located in the phospholipid bilayer and is hydrophobic itself, the release of CB caused by this effect is less obvious compared to that of BPA.

### 2.5. In Vitro Cell Experiments

#### 2.5.1. Cell Viability Assay

**Figure 7:**
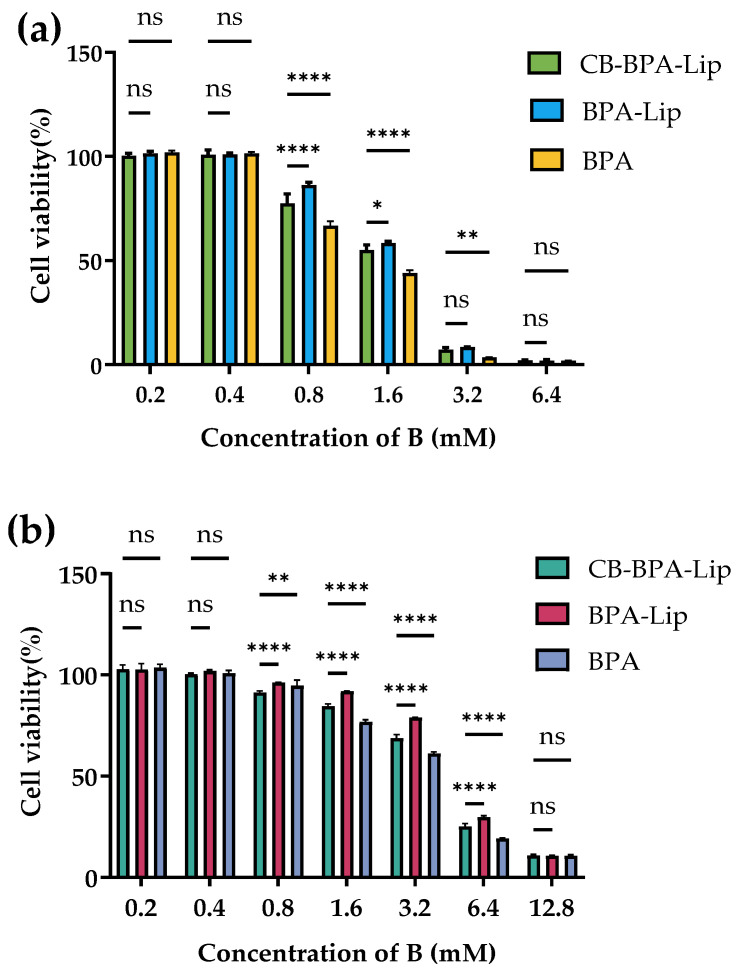
Cell viability assay. (**a**) Cell viability of A549 cells post-treatment with varied concentrations of B (*n* = 4); (**b**) cell viability of BEAS-2B cells post-treatment with varied concentrations of B (*n* = 4). B, boron; IC_50_, half-maximal inhibitory concentration. “ns” stands for not significant, * *p* ≤ 0.05, ** *p* ≤ 0.01, and **** *p* ≤ 0.0001. All data are an average of four independent experiments.

BNCT requires boron agents with low cytotoxicity. The Cell Counting Kit (CCK)-8 assay was therefore used to compare the effects of CB-BPA-Lips, BPA-Lips, and BPA on cell viability across boron concentrations. For A549 cells, the IC50 values were 1.564 mM B for CB-BPA-Lips, 1.710 mM B for BPA-Lips, and 1.274 mM B for BPA (Figure 7a). As for BEAS-2B cells, the IC50 values were 4.195 mM B for CB-BPA-Lips, 4.602 mM B for BPA-Lips, and 3.494 mM B for BPA (Figure 7b), indicating that both liposomal formulations impair viability less than BPA.

Both liposomal formulations exhibited a lower inhibitory effect on cell viability compared to BPA (BPA is generally considered to have relatively low cytotoxicity). In both A549 cells and BEAS-2B cells, the IC_50_ values of CB-BPA-Lips were higher than those of the BPA control group, indicating that the former had lower cytotoxicity. This may be related to the way liposomes enter cells via endocytosis, which significantly reduces the chance of direct contact between cells and the drug. Notably, when incubated with 0.8 mM B, all three test samples exhibited initial inhibitory effects on cell viability in both A549 and BEAS-2B cells. This might be attributed to the slow-release property of liposomal formulations. Another reason is that there was still a certain amount of boron-containing agents released from the liposomes during the 24 h incubation. This is worthy of attention. Even though the IC_50_ values of the two liposomal formulations are higher than those of BPA, it also suggests the importance of reducing the non-targeted release of liposomes in the future.

#### 2.5.2. Cellular Uptake Assay

**Figure 8:**
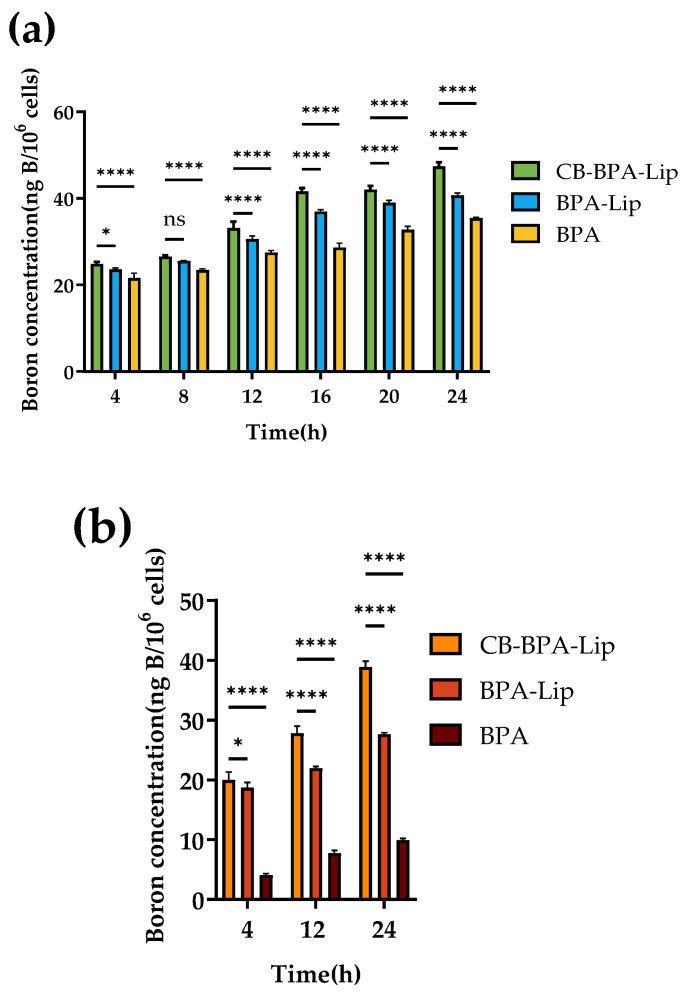
Cellular uptake assay. (**a**) Cellular uptake in A549 cells (*n* = 4); (**b**) cellular uptake in BEAS-2B cells (*n* = 4). “ns” stands for not significant, * *p* ≤ 0.05, and **** *p* ≤ 0.0001. All data are an average of four independent experiments.

In vitro boron uptake by tumor cells serves as a crucial metric for evaluating the potential of CB-BPA-Lips in further investigations of BNCT. To assess CB-BPA-Lips, CB-BPA-Lips, BPA-Lips, and BPA were co-incubated with A549 cells for 0–24 h at a boron concentration that had no significant impact on cell viability (0.4 mM B). After 24 h, the cellular boron content in the CB-BPA-Lips group (47.3642 ng B/10^6^ cells) was higher than that in the BPA-Lips control group (40.6753 ng B/10^6^ cells) and the BPA control group (35.4402 ng B/10^6^ cells) (Figure 8a) [22,23]. Boron uptake from CB-BPA-Lips, BPA-Lips, and BPA was measured in nonmalignant cells. To avoid confounding by LAT1-mediated BPA uptake, BEAS-2B cells were selected, which express low LAT1 and are commonly used as a LAT1-negative control [24]. Within 0–24 h, the samples were co-incubated with BEAS-2B cells at a boron concentration that had no significant impact on cell viability (0.4 mM B). The cells were then collected, digested, and measured at designated time points. After 24 h, the cellular boron content in the CB-BPA-Lips group was higher at 38.8875 ng B/10^6^ cells compared to the BPA-Lips control group at 27.6800 ng B/10^6^ cells and the BPA control group at 9.9220 ng B/10^6^ cells (Figure 8b). It can be found that the incorporation of CB into liposomes effectively increases the cellular boron uptake, and all the above results indicate that CB-BPA-Lips warrant future in vivo validation.

Higher uptake of CB-BPA-Lips was observed in both A549 tumor cells and BEAS-2B non-tumor cells with low LAT1 expression. Compared with the BPA control group, even in BEAS-2B cells, the low expression of LAT1 did not lead to a decrease in cellular boron uptake, which proves that the cellular boron uptake mainly originates from the endocytosis of boron-loaded liposomes. Although these results appear to be favorable, the in vitro cell results nonetheless raised concern: they implied that CB-BPA-Lips might not sufficiently discriminate between the intended target (tumor) and non-target tissue (normal) in vivo, potentially affecting the application of CB-BPA-Lips. Therefore, for in vivo experiments, in the future, attention should be paid to the boron enrichment in the targeted and non-targeted sites.

#### 2.5.3. Flow Cytometry

**Figure 9:**
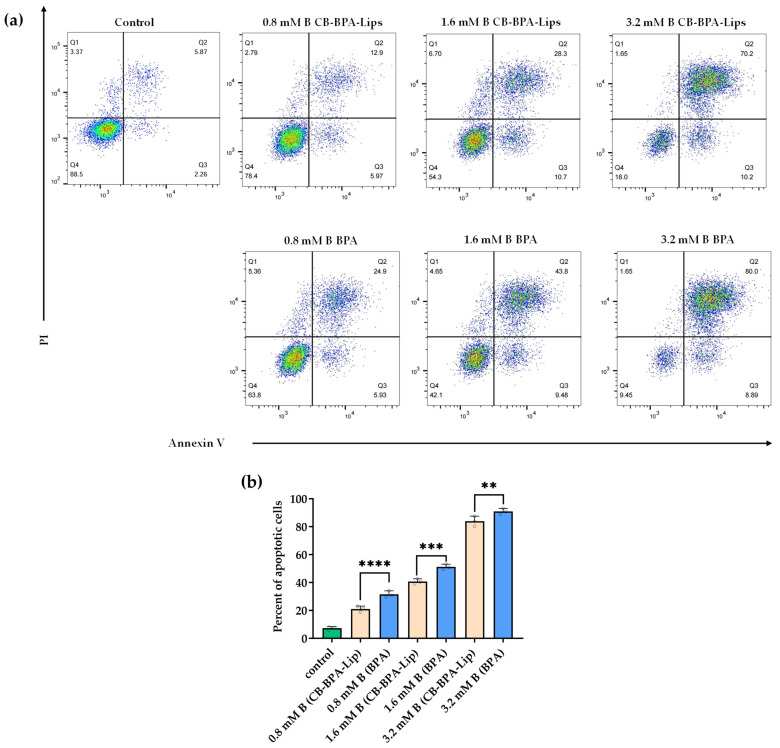
Flow cytometry. (**a**,**b**) Annexin V/PI staining for detection of apoptosis/necrosis in A549 cells administered with different boron concentrations (*n* = 3). ** *p* ≤ 0.01, *** *p* ≤ 0.001, and **** *p* ≤ 0.0001. All data are an average of three independent experiments.

Apoptosis in A549 cells was assessed by flow cytometry using Annexin V-FITC/PI staining. Annexin V detects phosphatidylserine exposed on the outer leaflet of the plasma membrane during early apoptosis, whereas propidium iodide (PI) intercalates with DNA and only enters cells once membrane integrity is lost, as in late apoptosis or necrosis. Thus, Annexin V/PI staining distinguishes early apoptotic, late apoptotic, and necrotic cells. Apoptosis induced by varying boron concentrations of CB-BPA-Lips or BPA in A549 cells was compared (Figure 9). Both CB-BPA-Lips and BPA induced early and late apoptosis at the boron concentrations tested, but CB-BPA-Lips produced less apoptosis than BPA across those concentrations.

## 3. Discussion

This study aims to propose a novel delivery system for BPA and a method for process optimization that may enhance the development of BNCT. Since the introduction of BNCT in the 1950s, various methods for delivering boron to tumor cells have been investigated [5,25,26,27]. To date, BPA remains the only boron agent approved for marketing. However, the high dosage of BPA, which may lead to increased economic costs for treatment, along with its short circulation time in the body and limited retention time in tumors, has hindered its further advancement. Lips, recognized as a widely studied drug delivery system, can effectively mitigate these challenges. Various nanoparticulate systems for BPA delivery in BNCT—such as liposomes [28], polymeric micelles [29], and multifunctional vesicles [30]—have been reported. Compared with nanocarriers whose syntheses are complex, Lips stand out as the most promising platform for near-term clinical translation for their obvious advantages. Decades of research on liposomes have produced extensive data on biocompatibility, in vivo pharmacokinetics, safety, and scalable manufacturing [31], thereby creating a strong theoretical and practical foundation for boron agent development. We argue that refining and optimizing well-characterized material systems already understood is often faster and more reliable than pursuing entirely new materials. Consequently, improving encapsulation efficiency in existing BPA–liposome formulations is a critical objective and one of the primary motivations of this study. In this study, we developed a novel BPA liposome by inserting CB—a boron-rich small molecule—into the phospholipid bilayer for BNCT, aiming to assist in breaking through the limitations of the research in the field of BPA liposomes.

Previous studies have mainly focused on the delivery of boron agents with limited targeting capabilities, such as BSH [32], CB [33], and their derivatives [34,35,36], and these studies have achieved good results in AsPC-1 human metastatic pancreatic adenocarcinoma, B16 melanoma mouse melanoma, or humanized EMT6 tumor. However, for BPA with tumor-targeting application value, there are relatively few reports related to its liposome delivery. During the research process, we found that two key challenges have led to a decline in researchers’ enthusiasm for BPA liposomes: one is that BPA has poor water solubility (requiring sorbitol or fructose as solubilizers), resulting in a low encapsulation efficiency [37]; second, the boron-carrying capacity is limited when only BPA is loaded into liposomes [38]. We did not give up this research direction but attempted to overcome these difficulties by incorporating boron-rich small molecule CB and optimizing the preparation conditions. These strategies were proven to be effective. The BBD response surface methodology was employed as a process optimization approach to investigate the effects of various factors on the encapsulation efficiency of BPA in CB-BPA-Lips. Through condition optimization and response surface design, the encapsulation efficiency of BPA increased from 19.64% (the result of the unoptimized group in this study) to 57.64%. This approach aims to increase the raw material utilization of BPA and provide valuable insights for the synthesis and large-scale production of diverse novel BPA liposomes [39]. Just as in previous studies on boron-containing liposomes, we conducted a series of characterizations on the novel liposome in this study, including morphology, zeta potential, hydrodynamic diameter, and PDI. It is worth noting, however, that we paid more attention to the stability study of liposome properties. Therefore, 50% FBS was used in the investigation of serum stability of CB-BPA-Lips. Previously, 10% FBS was usually used in the study of nanoparticle properties. However, considering that high-concentration FBS is closer to the in vivo physiological environment and that the property changes in liposomes caused by low-concentration FBS have high requirements for the precise detection of experimental equipment [40,41], we used the former and found through research that the properties of CB-BPA-Lips were stable for at least 48 h. As for the in vitro release experiment, we regarded it as part of the stability study, with the aim of exploring whether CB-BPA-Lips could prevent the rapid leakage of internally loaded drugs. The zero-order model, the first-order model, the Weibull model, the Higuchi model, and the Korsmeyer–Peppas model were introduced into this study, which more accurately described the drug release trends of BPA and CB in CB-BPA-Lips through an intuitive mathematical expression. Fortunately, both the drug release curve and the drug release kinetics model indicate that BPA and CB do not exhibit the problem of rapid leakage from CB-BPA-Lips. Regrettably, just like the vast majority of boron-containing liposomes at present, this study did not include the stimulus-responsive drug release mechanism in the physiological environment that is close to the tumor microenvironment. This strategy may enhance the therapeutic effect of BNCT in future studies [42] because the uniform dispersion of boron agents in tumor sites is of great significance for BNCT research. Therefore, in the subsequent work, we will take this strategy and the stability of the properties of the non-targeted sites of liposomes into account simultaneously.

Cell viability and apoptosis assays indicated that CB-BPA-Lips exhibited low cytotoxicity and favorable biocompatibility relative to BPA. This effect likely arises from the phospholipid bilayer of the liposomes, which either encapsulates the boron compound in the aqueous core or embeds it within the bilayer, thereby physically isolating the boron species from nonspecific interactions with the cell membrane and intracellular targets and preventing aberrant activation of apoptotic pathways [43,44]. Additionally, cellular uptake assays have shown that CB-BPA-Lips can effectively increase the boron uptake of cells compared with BPA-Lips, confirming the superiority of the co-loading strategy.

The original intention of our research was to use this preparation in the treatment of other sites in addition to the treatment of in situ lung cancer because the large volume of the lungs may affect the therapeutic effect of BNCT. Bone metastasis foci in the spine, pelvis, ribs, and proximal femur, where lung cancer is prone to metastasize, or adrenal metastasis are also within our initial scope of consideration. The construction of an ectopic tumor mouse model also provides a reference for further exploration of these ideas. Considering the limitations of BNCT in treating large cancerous organs, such as the liver and lung, the strategy of stimulus-responsive drug release is worthy of being incorporated into the research of boron-containing liposomes.

This study was confined to preliminary in vitro investigations. Although we have conducted a substantial number of in vitro experiments, the subsequent in vivo studies in animal models must encompass not only tumor boron concentration assessment, pharmacokinetic evaluation, and neutron irradiation assay but also the in vivo stability of liposomes, boron accumulation in both tumor and non-tumor sites, and tissue damage in non-tumor regions. These studies are essential to systematically investigate the potential applications of CB-BPA-Lips.

Furthermore, the liposomal interior also offers further development potential; it can host high-loading formulations of diverse agents, including chemotherapy, immunotherapy, photodynamic, and photothermal agents to pursue synergistic antitumor effects. The liposome surface is readily modifiable, enabling conjugation of targeting moieties. Prior work has shown that boron-containing liposomes modified with ligands such as folic acid (FA) [45], epidermal growth factor (EGF) [46], anti-carcinoembryonic antigen (CEA) [47], and arginine–glycine–aspartic acid (RGD) [48] possess promising applicability. These modification strategies may enhance the uptake of boron-loaded liposomes, thereby increasing the boron concentration in tumor cells while reducing boron accumulation in normal cells. This could contribute to BNCT achieving targeted tumor killing in lung cancer and other malignancies while sparing normal tissues. Subsequent investigations will focus on further in vivo studies of CB-BPA-Lips and the development of more innovative BPA liposomes. We will also incorporate these targeting strategies and multiple drug-loading formulations in future studies to enhance boron uptake in tumors and improve antitumor efficacy.

## 4. Materials and Methods

### 4.1. Materials and Reagents

Methanol of chromatographic grade, chloroform, and ether were obtained from Aladdin (Shanghai, China). Egg phosphatidylcholine (Egg PC), cholesterol, and 1,2-Distearoyl-sn-Glycero-3-Phosphoethanolamine-Polyethylene Glycol (DSPE-PEG_2000_) were purchased from Shanghai Aladdin (Shanghai, China). D-sorbitol was purchased from Macklin (Shanghai, China).

Fetal bovine serum was purchased from Beyotime Biotech Inc. (Shanghai, China). ^11^B-Boronophenylalanine (BPA) was purchased from Sigma-Aldrich (St. Louis, MO, USA) and ^11^B-o-carborane (CB) was purchased from Alfa Aesar (Shanghai, China). All the free BPA solutions used in this study were prepared in the laboratory using the following procedure: weigh an appropriate amount of D-sorbitol and BPA into a beaker to achieve a D-sorbitol: BPA mass ratio of 1.05:1. Add ultrapure water and adjust the pH to 9.5–10.0 with 1.0 M NaOH. Vortex until all solids dissolve. Then, lower the pH to 7.4 with 1 M hydrochloric acid and continue vortexing for at least 10 min.

### 4.2. Preparation of CB-BPA-Lips

CB-BPA-Lips were formulated from Egg PC, cholesterol, and DSPE-PEG2000 (mass ratio = 2:1:0.38) using the reverse-phase evaporation method [14]. Briefly, the lipids and CB were co-dissolved in 4 mL of chloroform/diethyl ether (1:1, *v*/*v*), followed by the addition of 1 mL of BPA–sorbitol solution (approximately 30 mg/mL). The mixture was then sonicated to form a water-in-oil emulsion, and the organic solvents were evaporated under reduced pressure at 45 °C, resulting in the formation of a gel. This gel was hydrated with phosphate-buffered saline at 45 °C, and the resulting Lips were passed through an Avanti Mini-Extruder (Avanti Mini-Extruder, Merck, Darmstadt, Germany) to ensure a homogeneous size distribution (the liposomes passed through the 100-nanometer membrane 10 times). At 4 °C and 2000 RPM for 15 min, free BPA was removed using a 50 kDa ultrafiltration centrifugal tube by ultra-low temperature centrifuge (Sorvall Legend Micro 21R, Thermo Fisher Scientific Inc., Waltham, MA, USA).

### 4.3. EE Calculation

The BPA EE was determined by ultrafiltration centrifugation (the recovery rate was determined by mixing free BPA with an equal volume of blank liposomes to prove that the recovery rate was greater than 98%). Separating free BPA via ultrafiltration from CB-BPA-Lips, an equal volume of CB-BPA-Lips was ultrasonically treated with 5 times methanol, and then analyzed using high-performance liquid chromatography (HPLC;LC-20A, SHIMADZU, Kyoto, Japan) at 210 nm. An isocratic mobile phase of methanol–0.1% phosphoric acid aqueous solution (4:96, *v*/*v*) was used at a flow rate of 1.0 mL/min, and the stationary phase was a ShimNex CS C18 column (250 × 4.6 mm, 5 μm, SHIMADZU, Kyoto, Japan). The BPA EE was calculated using Equation (2):

(2) $$\mathrm{EE}(\%)=(W_{\mathrm{total}}-W_{\mathrm{un}})/W_{\mathrm{total}}\times 100\%$$

where $W_{\mathrm{total}}$ represents the total BPA amount in liposome preparation, and $W_{\mathrm{un}}$ denotes the unencapsulated BPA amount in liposomes.

### 4.4. Experimental Design

A BBD with three factors, namely Egg PC-to-cholesterol mass ratio (1:1–3:1), Egg PC-to-BPA mass ratio (4:1–6:1), and hydration time (12–24 min), was employed to optimize variables affecting the BPA EE (Table 4). A total of 17 experimental runs were performed as part of the RSM [49]. The model equation describing the influence of the three independent variables on the response Y is given by Equation (3):

(3) $$Y=\beta_{0}+\sum_{i=1}^{3}\beta_{i}X_{i}+\sum_{i=1}^{3}\beta_{\mathrm{ii}}X_{i}^{2}+\sum_{i=1}^{2}\sum_{j=2}^{3}\beta_{\mathrm{ji}}X_{i}X_{j}$$

where Y represents the response variable, with $\beta_{0},\beta_{i},\beta_{\mathrm{ii}},\beta_{\mathrm{ji}}$ denoting the coefficients for the intercept, linear, quadratic, and interaction terms. $X_{i}$ and $X_{j}$ represent the independent variables.

### 4.5. Characterization

To determine the hydrodynamic diameter, PDI, and zeta potential of Lips, Lips at 5 mM lipid concentration were diluted at 0.2 mM with PBS (pH = 7.4). The hydrodynamic diameter, PDI, and zeta potential were measured through dynamic light scattering analysis (DLS; Zetasizer Nano ZS, Malvern Instruments Ltd., Malvern, UK), and morphology was examined by transmission electron microscopy (TEM; JEM-F200, JEOL Ltd., Tokyo, Japan). The samples were diluted several ten-fold, ultrasonicated for 1 min, and then stained with 0.5% uranyl acetate prior to analysis, following the previously described protocol [50].

### 4.6. In Vitro Serum Stability Assay

CB-BPA-Lips at 5 mM lipid concentration were diluted with PBS (pH = 7.4) containing FBS. Then, the CB-BPA-Lips were incubated at 2.5 mM lipid concentration in 50% FBS. The stability of liposome properties of CB-BPA-Lips in serum was evaluated by observing changes in hydrodynamic diameter, PDI, and zeta potential for 12, 24, and 48 h at 37 °C. The samples were diluted with bi-distilled water (1/50 *v*/*v*) and analyzed by DLS after sampling.

### 4.7. In Vitro Release Assay

To investigate the drug release kinetics, a dialysis-based assay was employed. Samples (2 mL) of CB-BPA-Lips were sealed in 50 kDa dialysis sacks and immersed in 100 mL of PBS (pH 7.4) containing 10% Tween 80. The dissolution vessels were then placed in a shaker (THZ-D, Changzhou Guoyu Instrument Manufacturing Co., Ltd., Changzhou, China) maintained at 37 °C and 100 rpm for 24 h. At 0.25–24 h, the medium was sampled and filtered for inductively coupled plasma mass spectrometry (ICP-MS, Agilent 7800, Agilent Technologies, Inc., Santa Clara, CA, USA) and HPLC (the concentration of CB at each time point was calculated by the difference between the total boron concentration measured by ICP-MS and the BPA concentration measured by HPLC; the feasibility of this method was verified by measuring the recovery rate after mixing CB with different boron concentrations (high, medium, and low) with BPA, as shown in Table S1), after which it was entirely replenished with fresh PBS containing 10% Tween 80 to ensure consistent sink conditions throughout the experiment.

### 4.8. In Vitro Cell Experiments

#### 4.8.1. Cell Culture Method

The A549 cell lines were obtained from Fuheng Biotechnology (Shanghai, China). All cell lines were cultured in F-12K medium (Kaighn’s Modification of Ham’s F-12 Medium) supplemented with 10% FBS, 1% penicillin, and streptomycin in 5% CO_2_ at 37 °C [51], and the medium was replaced every 1–2 days.

The BEAS-2B cell lines were obtained from Fuheng Biotechnology (Shanghai, China). All cell lines were cultured in DMEM medium supplemented with 10% FBS, 1% penicillin, and streptomycin in 5% CO_2_ at 37 °C, and the medium was replaced every 1–2 days.

#### 4.8.2. Cell Viability Assay

The CCK-8 assay was used to evaluate the effects of CB-BPA-Lips, BPA-Lips, and BPA on cell viability at different boron concentrations [52]. A549 or BEAS-2B cells (approximately 4 × 10^3^ per well) were seeded in 96-well plates and incubated at 37 ˚C overnight. The solutions containing CB-BPA-Lips, BPA-Lips, and BPA were diluted through the medium of the corresponding cell line (the medium contained 10% FBS, 1% penicillin, and streptomycin). Various concentrations of boron (0.2, 0.4, 0.8, 1.6, 3.2, and 6.4 mM B) were added to each well. After 24 h of culture, the medium was replaced with 200 uL of 10% CCK-8 solution, and incubation was halted after 2 h. Following 24 h of incubation with CCK-8, the absorbance at 450 nm was measured using a microplate reader [53] (Multiskan FC, Thermo Fisher Scientific Inc., Waltham, MA, USA), with cell-free wells as blanks. Cell viability was calculated using Equation (4):

(4) $${\mathrm{Cell}\mathrm{viability}(\%)=(\mathrm{OD}}_{\mathrm{test}}-{\mathrm{OD}}_{\mathrm{blank}})/({\mathrm{OD}}_{\mathrm{control}}-{\mathrm{OD}}_{\mathrm{blank}})\times 100\%$$

where ${\mathrm{OD}}_{\mathrm{test}}$ is the absorbance value of the test group, ${\mathrm{OD}}_{\mathrm{blank}}$ is the absorbance value of the blank group, and ${\mathrm{OD}}_{\mathrm{control}}$ is the absorbance value of the control group [54].

#### 4.8.3. Cellular Uptake Assay

Following an overnight incubation of A549 or BEAS-2B cells (1 × 10^5^ cells/well in a 24-well plate) at 37 °C, the culture was replaced with CB-BPA-Lips, BPA-Lips, and BPA (0.4 mM B) for specified durations (4–24 h). After treatment, cells were washed thrice with ice-cold PBS and lysed with a HNO_3_/H_2_O_2_ (4:1, *v*/*v*) solution. The obtained lysates were subjected to microwave-assisted digestion, diluted appropriately, and the intracellular boron content was directly determined by ICP-MS.

#### 4.8.4. Flow Cytometry

The types of cell death were detected using an Annexin V-FITC and PI staining kit (Merck, Germany). A549 cells co-incubated with different boron concentrations of CB-BPA-Lips or BPA for 24 h were collected by trypsin without EDTA (Beyotime Biotechnology, Shanghai, China). The cell suspension was centrifuged at 300× *g* for 5 min at 4 °C. The cells were washed twice with pre-cooled PBS and finally resuspended to a cell concentration of 1 × 10^6^–1 × 10^7^ cells/mL. Subsequently, 100 μL of the cell suspension containing approximately 1 × 10^5^ cells was taken, and then 5 μL of Annexin V-FITC was added and gently mixed, followed by incubation at room temperature in the dark for 15 min. Then, 5 μL of PI staining solution was added and mixed, and then incubated at room temperature in the dark for 5 min. Finally, 400 μL of Binding Buffer (1×, Beyotime Biotechnology, Shanghai, China) was added to resuspend the cells. The cells were analyzed using a flow cytometer (Attune Xenith, Thermo Fisher Scientific Inc., Waltham, MA, USA). An emission filter of 515–545 nm was used for FITC (green), and an emission filter of 600 nm was used for PI (red). The data were analyzed using FlowJo software version 10.90.

### 4.9. Statistical Analysis

All experiments were repeated at least three times unless noted otherwise. Statistical analyses were conducted with GraphPad Prism 10, and the data is presented as mean ± standard error of the mean. The *t*-test, one-way ANOVA, and two-way ANOVA were used to represent significant differences with the following notations: “ns” stands for not significant, * *p* < 0.05, ** *p* < 0.01, *** *p* < 0.001, and **** *p* < 0.0001.

## 5. Conclusions

In summary, we innovatively developed a liposomal formulation with simple materials and potentially lower costs (less BPA and CB without targeting modification) to co-deliver BPA and CB to supply boron to cancer cells for BNCT. We used BBD and RSM to optimize BPA loading and increase its concentration in the liposomes. In vitro release and serum-stability assays showed that CB-BPA-Lips had sustained-release behavior and robust stability. In A549 lung cancer cells, in vitro uptake reached 47.3642 ng B/10^6^ cells after 24 h, exceeding the uptake from BPA alone. Furthermore, we also found that CB-BPA-Lips with CB loaded in the phospholipid bilayer exhibited higher uptake by tumor cells compared to BPA-Lip. This may be related to the uptake of liposomes by tumor cells mediated via endocytosis or membrane fusion, as well as the better sustained-release ability of CB in the phospholipid bilayer of liposomes compared to BPA in the hydrophilic phase, as demonstrated by in vitro release studies. Consequently, more CB with limited tumor targeting ability can enter the cells. Altogether, the findings of this study highlight the potential of the developed liposomal formulation in enhancing boron availability and BNCT efficacy.

## Figures and Tables

**Table 1:** Box–Behnken design matrix and response values for the BPA EE. BPA, boronophenylalanine; EE, encapsulation efficiency.

	X_1_	X_2_	X_3_/min	Y (%)
1	−1	−1	0	38.050
2	1	−1	0	40.055
3	−1	1	0	42.648
4	1	1	0	42.661
5	−1	0	−1	42.301
6	1	0	−1	42.345
7	−1	0	1	41.036
8	1	0	1	43.267
9	0	−1	−1	43.493
10	0	1	−1	46.731
11	0	−1	1	42.525
12	0	1	1	45.919
13	0	0	0	56.334
14	0	0	0	55.991
15	0	0	0	56.100
16	0	0	0	56.775
17	0	0	0	56.408

**Table 2:** Results of ANOVA analysis.

Source	Sum of Squares	Degree of Freedom (DF)	Mean Square	F-Value	*p*-Value
model	724.70	9	80.52	830.28	<0.0001 ^a^
X_1_	2.30	1	2.30	23.75	0.0018 ^a^
X_2_	23.93	1	23.93	246.74	<0.0001 ^a^
X_3_	0.5634	1	0.5634	5.81	0.0468 ^a^
X_1_X_2_	0.9920	1	0.9920	10.23	0.0151 ^a^
X_1_X_3_	1.20	1	1.20	12.33	0.0098 ^a^
X_2_X_3_	0.0061	1	0.0061	0.0627	0.8094 ^b^
X_1_^2^	337.19	1	337.19	3476.89	<0.0001 ^a^
X_2_^2^	178.95	1	178.95	1845.16	<0.0001 ^a^
X_3_^2^	111.04	1	111.04	1144.99	<0.0001 ^a^
Residual	0.6789	7	0.0970		
Lack of fit	0.3073	3	0.1024	1.10	0.4455 ^b^
Pure error	0.3716	4	0.0929		
Cor. total	725.37	16			

R^2^ = 0.9991, Radj^2^ = 0.9979, and C.V.% = 0.6679%. ^a^ 5% significance level. ^b^ Not significant relative to the pure error.

**Table 3:** Drug release kinetic model parameters for CB-BPA-Lips. “Mt” denotes the cumulative drug released by time “t”. “M_∞_” denotes the theoretical maximum cumulative release. The ratio “Mt/M_∞_” is the cumulative release fraction. “R^2^” is the coefficient of determination. “k_0_”, “k_1_”, “k_h_”, and “k” are release rate constants. “n” is the diffusion index. “β” is the shape parameter. “τ” is the scale parameter.

	Drug Release Kinetic Model											
	Zero-Order		First-Order		Weibull			Higuchi		Korsmeyer–Peppas		
Samples	$\frac{M_{t}}{M_{\infty }}=k_{0}t$		$\ln(\begin{array}{c} 1-\frac{M_{t}}{M_{\infty }})=-k_{1}t \end{array}$		$M_{t}/M_{\infty }=1-\exp(-(t/\tau {)}^{\beta })$			$\frac{M_{t}}{M_{\infty }}=k_{h}t^{1/2}$		$\frac{M_{t}}{M_{\infty }}=kt^{n}$		
	R^2^	k_0_/% × h^−1^	R^2^	k_1_/h^−1^	R^2^	τ	β	R^2^	k_h_/% × h^−(1/2)^	R^2^	k/h^−n^	n
BPA	0.5897	0.93	0.8849	1.16	0.9934	2.2623	0.3336	0.7448	6.01	0.8704	43.06	0.14
CB	0.6189	1.21	0.9904	0.36	0.9917	0.3074	0.9408	0.8230	7.51	0.8807	15.08	0.32

**Table 4:** Analysis of variance. PC, phosphatidylcholine.

Independent Variables	Code	−1	0	1
Mass ratio of Egg PC to cholesterol	X_1_	1:1	2:1	3:1
Mass ratio of Egg PC to BPA	X_2_	4:1	5:1	6:1
Hydration time(min)	X_3_	12	18	24

## Data Availability

The original contributions presented in this study are included in the article/Supplementary Materials. Further inquiries can be directed to the corresponding author.

## Supplementary Materials

The following supporting information can be downloaded at: https://www.mdpi.com/article/10.3390/molecules31091409/s1. Table S1: Recovery rate of spiked samples.

## Abbreviations

The following abbreviations are used in this manuscript:

BNCT	Boron neutron capture therapy
BPA	Boronophenylalanine
CB	o-Carborane
Lips	Liposomes
EPR	Enhanced permeability and retention
BSH	Sodium borocaptate
PC	Phosphatidylcholine
DSPE-PEG_2000_	1,2-Distearoyl-sn-Glycero-3-Phosphoethanolamine-Polyethylene Glycol
EE	Encapsulation efficiency
B	Boron
PDI	Polydispersity index
BBD	Box–Behnken design
RSM	Response surface methodology
IC_50_	Half-maximal inhibitory concentration
TEM	Transmission electron microscopy
DLS	Dynamic light scattering analysis
HPLC	High-performance liquid chromatography
ICP-MS	Inductively coupled plasma mass spectrometry
FBS	Fetal bovine serum
PBS	Phosphate-buffered saline

## References

[1] Hu C.R., Lai Y.Q., Cao Y., Pan J.J., Liu Y.H., Teng Y.C., Xia X.B. (2025). MRI and 18F-BPA PET-guided targeting in boron neutron capture therapy. Sci. Rep..

[2] Miyatake S.I., Kawabata S., Goto H., Narita Y., Suzuki M., Hirose K., Takai Y., Ono K., Ohnishi T., Tanaka H. (2020). Accelerator-based BNCT in rescue treatment of patients with recurrent GBM: A multicenter phase II study. J. Clin. Oncol..

[3] Nomoto T., Inoue Y., Yao Y., Suzuki M., Kanamori K., Takemoto H., Matsui M., Tomoda K., Nishiyama N. (2020). Poly(vinyl alcohol) boosting therapeutic potential of p-boronophenylalanine in neutron capture therapy by modulating metabolism. Sci. Adv..

[4] Xu H., Liu J., Li R.X., Lin J.J., Gui L.J., Wang Y.X., Jin Z.Y., Xia W., Liu Y.H., Cheng S.J. (2024). Novel promising boron agents for boron neutron capture therapy: Current status and outlook on the future. Coord. Chem. Rev..

[5] Matovic J., Järvinen J., Sokka I.K., Imlimthan S., Aitio O., Sarparanta M., Rautio J., Ekholm F.S. (2024). Towards New Delivery Agents for Boron Neutron Capture Therapy: Synthesis and In vitro Evaluation of a Set of Fluorinated Carbohydrate Derivatives. Molecules.

[6] Simone M.I. (2023). Diastereoselective Synthesis of the Borylated d-Galactose Monosaccharide 3-Boronic-3-Deoxy-d-Galactose and Biological Evaluation in Glycosidase Inhibition and in Cancer for Boron Neutron Capture Therapy (BNCT). Molecules.

[7] Voinea M., Simionescu M. (2002). Designing of ‘intelligent’ liposomes for efficient delivery of drugs. J. Cell. Mol. Med..

[8] Hashida M. (2022). Advocation and advancements of EPR effect theory in drug delivery science: A commentary. J. Control. Release.

[9] Liu Y., Lin W., Yang Y., Shao J.J., Zhao H.Y., Wang G.R., Shen A.G. (2022). Role of cuproptosis-related gene in lung adenocarcinoma. Front. Oncol..

[10] Lee Y.H., Do S.K., Lee S.Y., Kang H.G., Choi J.E., Hong M.J., Lee J.H., Lee S.W., Lee W.K., Jeong J.Y. (2023). Genetic variants in histone modification regions predicts clinical outcomes of pemetrexed chemotherapy in lung adenocarcinoma. Oncology.

[11] Lin X.Q., Guo H., Zhao W., Li M., Lin G., Chu Q., Chen E., Chen L.A., Chen R., Chu T.Q. (2025). Expert consensus on cancer treatment-related lung injury. J. Thorac. Dis..

[12] Zhang J., Feng Y., Dai S.H., Xu G.Q., Cheng B., Jiang L., Xue L.P., Liang W.H., Tang J. (2025). Neoadjuvant Chemoimmunotherapy in Non-small-cell Lung Cancer Patients: Effects on Pulmonary Function and Incidence of Postoperative Pulmonary Complications-A Multicentre Real-World Study. Eur. J. Cardio-Thorac. Surg..

[13] Wallace N.D., Bressel M., Hardcastle N., McIntosh L., Bucknell N., Kron T., Callahan J., Hicks R., Ball D., Macmanus M. (2025). A prospective study of Gallium-68 ventilation and perfusion PET/CT during and after radiotherapy in patients with non-small cell lung cancer. Radiother. Oncol..

[14] Takeuchi I., Ishizuka Y., Uchiro H., Makino K. (2017). Detailed biodistribution of liposomes prepared with polyborane instead of cholesterol for BNCT: Effects of PEGylation. Colloid Polym. Sci..

[15] Feng B., Tomizawa K., Michiue H., Miyatake S., Han X.J., Fujimura A., Seno M., Kirihata M., Matsui H. (2009). Delivery of sodium borocaptate to glioma cells using immunoliposome conjugated with anti-EGFR antibodies by ZZ-His. Biomaterials.

[16] Kueffer P.J., Maitz C.A., Khan A.A., Schuster S.A., Shlyakhtina N.I., Jalisatgi S.S., Brockman J.D., Nigg D.W., Hawthorne M.F. (2013). Boron neutron capture therapy demonstrated in mice bearing EMT6 tumors following selective delivery of boron by rationally designed liposomes. Proc. Natl. Acad. Sci. USA.

[17] Fukumura M., Nonoguchi N., Kawabata S., Hiramatsu R., Futamura G., Takeuchi K., Kanemitsu T., Takata T., Tanaka H., Suzuki M. (2023). 5-Aminolevulinic acid increases boronophenylalanine uptake into glioma stem cells and may sensitize malignant glioma to boron neutron capture therapy. Sci. Rep..

[18] Kondo N., Takada S., Hagimori M., Temma T. (2023). Development of a 2-(2-Hydroxyphenyl)-1H-benzimidazole-Based Fluorescence Sensor Targeting Boronic Acids for Versatile Application in Boron Neutron Capture Therapy. Cancers.

[19] Wang Y.Y., Wang M., Lin F., Zhang X.Y., Zhao Y.M., Guo C.Y., Wang J. (2022). Preparation, Characterization, and Evaluation of Liposomes Containing Oridonin from Rabdosia rubescens. Molecules.

[20] Soliman N.M., Shakeel F., Haq N., Alanazi F.K., Alshehri S., Bayomi M., Alenazi A.S.M., Alsarra I.A. (2022). Development and Optimization of Ciprofloxacin HCl-Loaded Chitosan Nanoparticles Using Box-Behnken Experimental Design. Molecules.

[21] Alam P., Siddiqui N.A., Rehman M.T., Hussain A., Akhtar A., Mir S.R., Alajmi M.F. (2021). Box-Behnken Design (BBD)-Based Optimization of Microwave-Assisted Extraction of Parthenolide from the Stems of *Tarconanthus camphoratus* and Cytotoxic Analysis. Molecules.

[22] Chen Y., Cheng G., Mahato R.I. (2008). RNAi for treating hepatitis B viral infection. Pharm. Res..

[23] Tomitaka A., Arami H., Huang Z., Raymond A., Rodriguez E., Cai Y., Febo M., Takemura Y., Nair M. (2017). Hybrid magneto-plasmonic liposomes for multimodal image-guided and brain-targeted HIV treatment. Nanoscale.

[24] Le Vee M., Jouan E., Lecureur V., Fardel O. (2016). Aryl hydrocarbon receptor-dependent up-regulation of the heterodimeric amino acid transporter LAT1 (*SLC7A5*)/CD98hc (*SLC3A2*) by diesel exhaust particle extract in human bronchial epithelial cells. Toxicol. Appl. Pharmacol..

[25] Seneviratne D.S., Saifi O., Mackeyev Y., Malouff T., Krishnan S. (2023). Next-Generation Boron Drugs and Rational Translational Studies Driving the Revival of BNCT. Cells.

[26] Li X., Liu Z., Liu K., Wang C., Liu Z., Xu X. (2026). Advances in Clinical Trials of Boron Neutron Capture Therapy. Research.

[27] Monti Hughes A., Hu N. (2023). Optimizing Boron Neutron Capture Therapy (BNCT) to Treat Cancer: An Updated Review on the Latest Developments on Boron Compounds and Strategies. Cancers.

[28] Proshkina G.M., Shramova E.I., Mirkasymov A.B., Zavestovskaya I.N., Deyev S.M. (2025). Targeted Nanoliposomes for the Delivery of Boronophenylalanine into HER2-Positive Cells. Acta Naturae.

[29] Huang L.C.S., Hsieh W.Y., Chen J.Y., Huang S.C., Chen J.K., Hsu M.H. (2014). Drug delivery system design and development for boron neutron capture therapy on cancer treatment. Appl. Radiat. Isot..

[30] Dai L.Q., Liu J., Yang T.Y., Yu X.R., Lu Y., Pan L.L., Zhou S.M., Shu D.Y., Liu Y.H., Mao W.Y. (2025). Lipoic acid-boronophenylalanine-derived multifunctional vesicles for cancer chemoradiotherapy. Nat. Commun..

[31] Ci T.Y., Zhang W.T., Qiao Y.Y., Li H.J., Zang J., Li H.J., Feng N.A.P., Gu Z. (2022). Delivery strategies in treatments of leukemia. Chem. Soc. Rev..

[32] Mehta S.C., Lai J.C.K., Lu D.R. (1996). Liposomal formulations containing sodium mercaptoundecahydrododecaborate (BSH) for boron neutron capture therapy. J. Microencapsul..

[33] Feakes D.A., Shelly K., Hawthorne M.F. (1995). Selective Boron Delivery To Murine Tumors By Lipophilic Species Incorporated In The Membranes of Unilamellar Liposomes. Proc. Natl. Acad. Sci. USA.

[34] Yanagie H., Tomita T., Kobayashi H., Fujii Y., Takahashi T., Hasumi K., Nariuchi H., Sekiguchi M. (1991). Application of Boronated Anti-Cea Immunoliposome to Tumor-Cell Growth-Inhibition In Invitro Boron Neutron-Capture Therapy Model. Br. J. Cancer.

[35] Shelly K., Feakes D.A., Hawthorne M.F., Schmidt P.G., Krisch T.A., Bauer W.F. (1992). Model Studies Directed Toward The Boron Neutron-Capture Therapy of Cancer–Boron Delivery To Murine Tumors With Liposomes. Proc. Natl. Acad. Sci. USA.

[36] Takeuchi I., Kanno Y., Uchiro H., Makino K. (2019). Polyborane-encapsulated PEGylated Liposomes Prepared Using Post-insertion Technique for Boron Neutron Capture Therapy. J. Oleo Sci..

[37] Luderer M.J., Muz B., Alhallak K., Sun J., Wasden K., Guenthner N., de la Puente P., Federico C., Azab A.K. (2019). Thermal Sensitive Liposomes Improve Delivery of Boronated Agents for Boron Neutron Capture Therapy. Pharm. Res..

[38] Pavanetto F., Perugini P., Genta I., Minoia C., Ronchi A., Prati U., Roveda L., Nano R. (2000). Boron-loaded liposomes in the treatment of hepatic metastases: Preliminary investigation by autoradiography analysis. Drug Deliv..

[39] Yang B., Lu F.Y., Li P.P., Ma J.J., Yang J., Zhang X.X., Cheng M., Yu W.J., Chai Y., Zou Y. (2025). An efficient measure for the isolation of chenodeoxycholic acid from chicken biles using enzyme-assisted extraction and macroporous resins refining. Poult. Sci..

[40] Kuai R., Yuan W.M., Li W.Y., Qin Y., Tang J., Yuan M.Q., Fu L., Ran R., Zhang Z.R., He Q. (2011). Targeted Delivery of Cargoes into a Murine Solid Tumor by a Cell-Penetrating Peptide and Cleavable Poly(ethylene glycol) Comodified Liposomal Delivery System via Systemic Administration. Mol. Pharm..

[41] Paun R.A., Dumut D.C., Centorame A., Thuraisingam T., Hajduch M., Mistrik M., Dzubak P., De Sanctis J.B., Radzioch D., Tabrizian M. (2022). One-Step Synthesis of Nanoliposomal Copper Diethyldithiocarbamate and Its Assessment for Cancer Therapy. Pharmaceutics.

[42] Lopez A., Holbrook J.H., Kemper G.E., Lukowski J.K., Andrews W.T., Hummon A.B. (2024). Tracking Drugs and Lipids: Quantitative Mass Spectrometry Imaging of Liposomal Doxorubicin Delivery and Bilayer Fate in Three-Dimensional Tumor Models. Anal. Chem..

[43] Gifford I., Vreeland W., Grdanovska S., Burgett E., Kalinich J., Vergara V., Wang C.K.C., Maimon E., Poster D., Al-Sheikhly M. (2014). Liposome-based delivery of a boron-containing cholesteryl ester for high-LET particle-induced damage of prostate cancer cells: A boron neutron capture therapy study. Int. J. Radiat. Biol..

[44] Ferrer-Ugalde A., Muñoz-Juan A., Laromaine A., Curotto P., Nievas S., Dagrosa M.A., Couto M., Núñez R. (2025). Enhancing Boron Neutron Capture Therapy (BNCT) with Materials Based on COSAN-Functionalized Nanoparticles. Pharmaceuticals.

[45] Singh A., Kim B.K., Mackeyev Y., Rohani P., Mahajan S.D., Swihart M.T., Krishnan S., Prasad P.N. (2019). Boron-Nanoparticle-Loaded Folic-Acid-Functionalized Liposomes to Achieve Optimum Boron Concentration for Boron Neutron Capture Therapy of Cancer. J. Biomed. Nanotechnol..

[46] Kullberg E.B., Bergstrand N., Carlsson J., Edwards K., Johnsson M., Sjöberg S., Gedda L. (2002). Development of EGF-conjugated liposomes for targeted delivery of boronated DNA-binding agents. Bioconjugate Chem..

[47] Yanagie H., Tomita T., Kobayashi H., Fujii Y., Nonaka Y., Saegusa Y., Hasumi K., Eriguchi M., Kobayashi T., Ono K. (1997). Inhibition of human pancreatic cancer growth in nude mice by boron neutron capture therapy. Br. J. Cancer.

[48] Koning G.A., Fretz M.M., Woroniecka U., Storm G., Krijger G.C. (2004). Targeting liposomes to tumor endothelial cells for neutron capture therapy. Appl. Radiat. Isot..

[49] Liu S., Ho P.C. (2017). Formulation optimization of scutellarin-loaded HP-β-CD/chitosan nanoparticles using response surface methodology with Box-Behnken design. Asian J. Pharm. Sci..

[50] Guo R., Deng M., He X., Li M., Li J., He P., Liu H., Li M., Zhang Z., He Q. (2022). Fucoidan-functionalized activated platelet-hitchhiking micelles simultaneously track tumor cells and remodel the immunosuppressive microenvironment for efficient metastatic cancer treatment. Acta Pharm. Sin. B.

[51] Zhang R.-Y., Liu Z.-K., Wei D., Yong Y.-L., Lin P., Li H., Liu M., Zheng N.-S., Liu K., Hu C.-X. (2021). UBE2S interacting with TRIM28 in the nucleus accelerates cell cycle by ubiquitination of p27 to promote hepatocellular carcinoma development. Signal Transduct. Target. Ther..

[52] Kim H.Y., Cho S., Kim S.B., Song E.C., Jung W., Shin Y.G., Suh J.H., Choi J., Yoon I., Kim U. (2024). Specific targeting of cancer vaccines to antigen-presenting cells via an endogenous TLR2/6 ligand derived from cysteinyl-tRNA synthetase 1. Mol. Ther..

[53] Sun L., He L., Wu W., Luo L., Han M., Liu Y., Shi S., Zhong K., Yang J., Li J. (2021). Fibroblast membrane-camouflaged nanoparticles for inflammation treatment in the early stage. Int. J. Oral Sci..

[54] Li M., Liu B., Xu W., Zhao L., Wang Z., He H., Li J., Wang F., Ma C., Liu K. (2023). Engineering Protein Coacervates into a Robust Adhesive for Real-Time Skin Healing. Engineering.

