# Supplementary material for: Process Optimization of Novel Boronophenylalanine Liposomes Through Box–Behnken Response Surface Design and Preliminary Evaluation in A549 Lung Carcinoma Cells for Boron Neutron Capture Therapy
Source: Molecules. 2026 Apr 24;31(9):1409. doi: 10.3390/molecules31091409 (PMC13165006; doi:10.3390/molecules31091409)
Supplement: Supplementary file 1 [file molecules-31-01409-s001.zip › molecules-4159977-supplementary.pdf]

Table S1. Recovery rate of spiked samples (n = 3). CB, carborane; BPA, boronophenylalanine; RSD, relative Standard Deviation.

Theoretical boron concentration of CB (μg/mL)	Theoretical total boron concentration (μg/mL)	Measured boron concentration of BPA (μg/mL)	Measured total boron concentration (μg/mL)	Measured boron concentration of CB (μg/mL)	Recovery rate (%)	Mean value (%)	RSD (%)
		75.6665	100.4140	24.7475	98.99		
25	100	75.6545	100.2245	24.5700	98.28	99.2241	1.0888
		75.3435	100.4441	25.1006	100.4024		
		50.5542	100.1321	49.5779	99.1558		
50	100	50.3404	100.1255	49.7851	99.5702	99.8707	0.9047
		49.999	100.4421	50.4431	100.8862		
		25.3247	100.3251	75.0004	100.0005		
75	100	25.9264	100.4251	74.4987	99.3316	99.5905	0.3606
		25.5529	100.1324	74.5795	99.4393		
